# Development of an OP9 Derived Cell Line as a Robust Model to Rapidly Study Adipocyte Differentiation

**DOI:** 10.1371/journal.pone.0112123

**Published:** 2014-11-19

**Authors:** Jacqueline M. Lane, Jamie R. Doyle, Jean-Philippe Fortin, Alan S. Kopin, José M. Ordovás

**Affiliations:** 1 Massachusetts General Hospital, Center for Human Genetics Research, 185 Cambridge Street, Boston, MA 02114, United States of America; 2 Division of Sleep Medicine, Brigham and Women's Hospital, 75 Francis St, Boston, MA 02115, United States of America; 3 Jean Mayer-USDA Human Nutrition Research Center on Aging at Tufts University, Boston, MA 02111, United States of America; 4 Tufts Medical Center, Molecular Cardiology Research Institute, Molecular Pharmacology Research Center, 800 Washington St., Box 7703, Boston, MA 02111, United States of America; Baylor College of Medicine, United States of America

## Abstract

One hallmark of obesity is adipocyte hypertrophy and hyperplasia. To gain novel insights into adipose biology and therapeutics, there is a pressing need for a robust, rapid, and informative cell model of adipocyte differentiation for potential RNAi and drug screens. Current models are prohibitive for drug and RNAi screens due to a slow differentiation time course and resistance to transfection. We asked if we could create a rapid, robust model of adipogenesis to potentially enable rapid functional and obesity therapeutic screens. We generated the clonal population OP9-K, which differentiates rapidly and reproducibly, and displays classic adipocyte morphology: rounded cell shape, lipid accumulation, and coalescence of lipids into a large droplet. We further validate the OP9-K cells as an adipocyte model system by microarray analysis of the differentiating transcriptome. OP9-K differentiates via known adipogenic pathways, involving the transcriptional activation and repression of common adipose markers Plin1, Gata2, C/Ebpα and C/Ebpβ and biological pathways, such as lipid metabolism, PPARγ signaling, and osteogenesis. We implemented a method to quantify lipid accumulation using automated microscopy and tested the ability of our model to detect alterations in lipid accumulation by reducing levels of the known master adipogenic regulator Pparγ. We further utilized our model to query the effects of a novel obesity therapeutic target, the transcription factor SPI1. We determine that reduction in levels of Spi1 leads to an increase in lipid accumulation. We demonstrate rapid, robust differentiation and efficient transfectability of the OP9-K cell model of adipogenesis. Together with our microscopy based lipid accumulation assay, adipogenesis assays can be achieved in just four days' time. The results of this study can contribute to the development of rapid screens with the potential to deepen our understanding of adipose biology and efficiently test obesity therapeutics.

## Introduction

Obesity is associated with an elevated risk of cardiovascular disease [1], diabetes [2], cancer [3], and other chronic diseases [4]–[8]. The physiology of obesity is characterized by an increase in adipocyte size (hypertrophy) and number (hyperplasia) [9]–[12]. In order to identify the genes and pathways essential for the development of adipocytes, we need a model system for rapid discoveries in adipose biology.

The process of adipocyte generation, adipogenesis, can be modeled in cell culture. In order to systemically identify the genes essential for adipogenesis, we need a model system which is amenable to RNAi and drug screening. Current models are prohibitive for rapid screens due to a slow differentiation time course, waning adipogenic culture with passage, and resistance to transfection. Gene knockdown (RNAi) screens are useful for identifying novel therapeutic targets and mapping disease pathways [13]. The effects of transient RNAi knockdown generally last approximately 96 hours, requiring a model of adipogenesis with a rapid differentiation period. As well, the cells must be readily transfectable. Lastly, an automated analysis method needs to be employed. The most widely used model of adipocyte biology is the 3T3-L1 cell line [14]–[16]. 3T3-L1 adipocytes differentiate over a period of twelve days with adipogenic stimuli [17], are difficult to transfect [18], and have waning adipogenic potential with passage [19]–[22]. Although advances have been made in high-throughput [23] and rapid [24] gene knock-down assays in adipogenesis, a method which is both rapid and high-throughput will greatly accelerate obesity therapeutic target discovery and treatment development.

To enable rapid advances in adipose biology, we generated a new clonal cell line utilizing OP9 cells, originally described by Wolins et al [25]. This cell line is a model of adipogenesis potentially suitable for high-throughput screening. OP9 cells are mouse bone marrow derived stromal cells that accumulate large triglyceride filled droplets after only 72 hours of adipogenic stimuli. OP9 cell differentiation is a PPARγ dependent process; differentiated cells express PPARγ, CEBPα, CEBPβ, PLIN1, and PLIN4 proteins similar to other adipogenesis models. OP9 cells are therefore a potential tool for rapid screening of adipogenesis.

In this paper we investigate the feasibility of OP9 clonal derived cells as a model for rapid screening of drug and gene knockdown effects on adipogenesis. This will enable a systematic analysis of the effects of single gene disruptions on mammalian adipogenesis in a cost-effective and rapid manner. First, we established a clonal population of OP9 cells, OP9-K, which differentiate rapidly, robustly, and reproducibly. We compared the transcriptome of differentiating OP9-K cells to other models of adipogenesis, and established the pathways through which OP9-K adipogenesis occurs. We also determined that OP9-K cells are amenable to transfection with an efficiency of>80%. Next, we developed a high-throughput microscopy assay for triglyceride quantification, enabling automated assessment of the progression of adipogenesis. As a proof of concept, we knocked-down the master adipogenic regulator Pparγ in OP9-K cells and confirmed inhibition of adipogenesis through our image-based assay. We utilized our model to query the effects of a novel obesity therapeutic target, the transcription factor SPI1. We conclude that a reduction in levels of Spi1 leads to an increase in lipid accumulation. Taken together these results show the potential to identify novel therapeutic targets and map disease pathways in a cost-effective, robust, and rapid fashion using differentiating OP9-K cells.

## Results

### Clonal OP9 cell lines differentiate rapidly and with high efficiency

To study the effect of gene knockdown on adipogenesis, we created a highly efficient preadipocyte clonal OP9 cell line. Previously, OP9 cells were shown to differentiate upon treatment with insulin oleate media [25]. Using the parental OP9 line and 4 clonal populations we tested triglyceride accumulation, lipophilic Nile Red stain accumulation, and lipid droplet formation.

**Figure 1:**
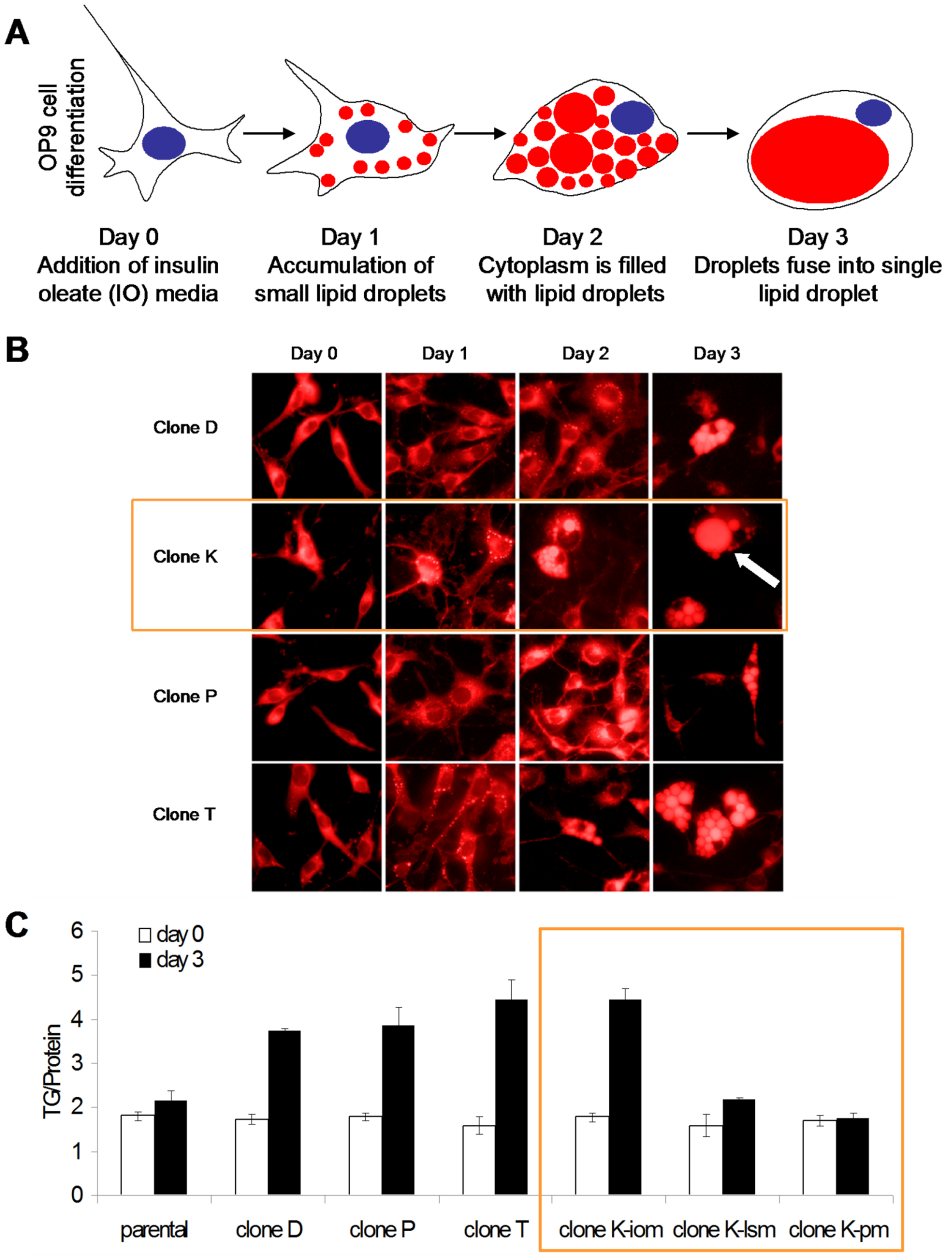
Selection of clonal preadipogenic cell line as a rapidly differentiating adipogenesis model. (A) Schematic display of robust OP9 adipogenesis demonstrating the desired morphological characteristics of a rounded cell, peripheral nucleus, lipid accumulation, and a coalesced lipid droplet. Triglyceride accumulation shown in red and nucleus shown in blue. OP9 cells differentiate over the course of 72 hours. After one day of insulin oleate (IO) media exposure, cells begin to accumulate small lipid droplets. After two days of IO media exposure, cells adopt a round morphology, the nucleus migrates to the periphery, and the cytoplasm is filled with lipid droplet. After three days of IO media exposure, cells resemble mature adipocytes. The nucleus is peripherally located and lipid droplets have fused into one large lipid droplet. OP9 cells are capable of differentiation at sub-confluent levels. (B) OP9 clonal populations were differentiated into adipocytes using insulin oleate (IO) media. Clone K (orange box) demonstrates desired adipogenesis morphology. Cells were fixed with paraformaldehyde and then stained with the lipophilic dye, Nile Red. Nile Red stain is shown in red. The arrow points to a mature adipocyte. Magnification is 40X. (C) Triglyceride (TG) content of clonal OP9 cell lines after 72 hours exposure to IO media, low serum media (lsm), or propagation media (pm), using TG assay. Assays performed in triplicate. Clone K (orange box) robustly accumulates triglyceride. Clone K consistently demonstrated adipocyte morphology of a rounded cell, coalesced lipid droplet, and triglyceride accumulation. Therefore, clone K OP9 cells were selected for further study.

During differentiation preadipocytes accumulate lipid droplets, which are filled with triglycerides. To assess the differentiation of OP9 cells, cells were treated with insulin oleate media (IO) for 72 hours and triglyceride levels were measured. Triglyceride content in parental OP9 cells increased 10% after 3 days in differentiation media (Fig 1c). OP9 cells were also stained with the lipophilic stain, Nile Red, to assess lipid content. Microscopy revealed <10% of the OP9 parental cells accumulate lipid droplets (data not shown).

In order to achieve robust differentiation we selected clonal OP9 cell lines. Interestingly, clonal populations grew at various rates and had varied morphology. Several clonal lines were assessed for lipid accumulation. Most of the clonal cell lines grew rapidly, had spindly morphology, and differentiated poorly (data not shown). Four clonal lines with increased lipid accumulation were selected for further study. After 3 days of IO media the cell lines increased triglycerides compared to the parental OP9 cell line (Fig 1b, c). Differentiated clone K OP9 cell lines accumulated 2-fold more triglyceride than undifferentiated clone K OP9 cells. Additionally all four clonal cells lines showed a significant increase in Nile Red staining between day 0 and day 3 cells (Fig 1b). After treatment with IO media all selected clonal cell lines visibly accumulate lipid droplets (Fig 1b). Differentiated clone K and T cells also display hallmark adipocyte morphology: large lipid droplets and a peripherally located nucleus (Figure 1b). Several clone K cells display a mature adipocyte phenotype consisting of one large coalesced lipid droplet (arrow in Figure 1b). Greater than 90% of OP9 clone K cells contain lipid droplets after 3 days in IO media, the potential for this phenotype does not wane with time. Therefore OP9 clone K cells were selected for further characterization.

### Response of the OP9 clone K transcriptome to adipogenesis

**Figure 2:**
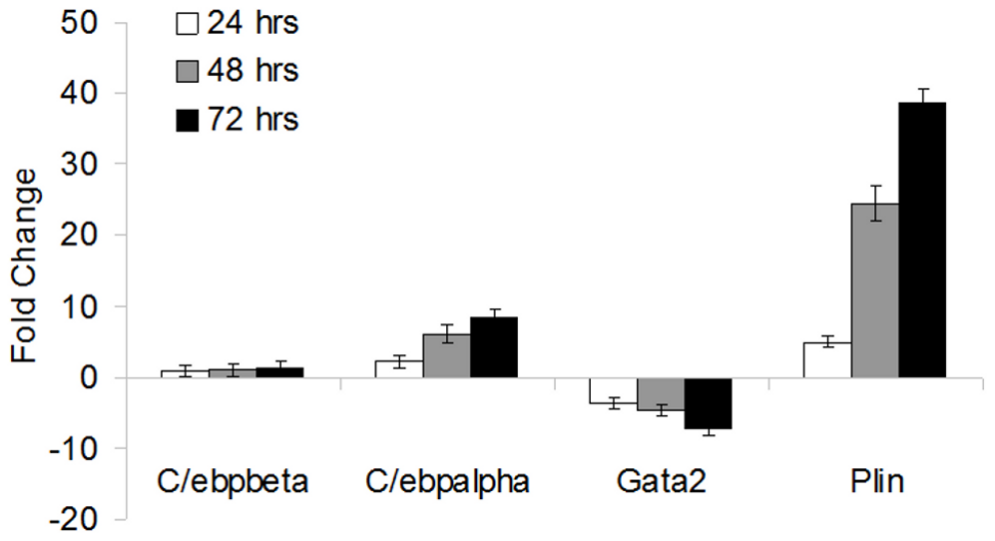
The transcriptome of OP9 clone K cells is analogous to previous models of adipogenesis. Mid and late stage known adipocyte markers are confirmed during OP9 cell adipogenesis. Quantitative PCR of adipocyte marker genes at 0, 24, 48, and 72 hrs after addition of IO media. Standard deviation is derived from 3 replicates.

To evaluate if OP9 cells undergo a differentiation process similar to other adipocyte models, a microarray was performed on RNA extracted from OP9 clone K cells during the course of differentiation (Appendices S2-S13 in Files S1 and S2). RNA from clone K cells treated for 0, 24, 48, or 72 hours with IO media was collected (corresponding to day 0, 1, 2, and 3). Samples were hybridized to the Affymetrix GeneChip Mouse Gene 1.0 ST Array and analyzed using Expression Console from Affymetrix and the R Limma package. To verify the results of the microarray, quantitative PCR (qPCR) was performed on four known adipose marker genes: Plin1, Gata2, C/Ebpα and C/Ebpβ (Figure 2). Microarray and qPCR demonstrate C/Ebpα and Plin1 are upregulated and Gata2 is downregulated during OP9 adipogenesis (Figure 2 and Table 1). C/Ebpβ is expressed in OP9 cells during adipogenesis, but levels do not significantly change during the course of differentiation. This corroborates protein expression data from Wolins et al. suggesting that OP9 cells are later stage preadipocytes [25]. Tables S1 and S2 show the 25 most up/down regulated genes from each time point.

**Table 1:** Microarray analysis profile of probes for adipocyte marker genes.

	Fold Change (p-value)		
Gene Symbol	Day 1	Day 2	Day 3
Plin1	6.88 (1.5E-10)	16.63 (2.75E-12)	21.81 (9.85E-13)
Cebpα	1.88 (1.7E-7)	2.89 (1.18E-9)	3.42 (1.36E-10)
Cebpβ	1.07 (0.200)	-1.04 (0.314)	-1.18 (0.002)
Gata2	−1.33 (0.008)	−1.5 (3.83E-4)	−1.79 (2.15E-5)

Expression values are RMA normalized and log transformed. Fold change is relative to Day 0.

To determine which biological processes are involved in OP9 adipogenesis, the 250 most differentially regulated genes at each time point were analyzed for pathway enrichment using GProfiler [26]. Adipose and bone cells share a common progenitor and differentiation is mutually exclusive [27]. In our samples we see confirmation of the interplay between bone and adipose differentiation; Day 1 OP9 cells down regulate cartilage development, signaling to bone morphogenic proteins, and proteinaceous extracellular matrix (Figure S1). Furthermore, adipocytes are mitotically quiescent cells [28], and we see down regulation of cell cycle components during day 2 and day 3 of OP9 adipogenesis. We also see down regulation of transcripts associated with miR 568, miR 374, and miR 590-3p.

During the entire differentiation time course the following processes are upregulated: fat cell differentiation, lipid metabolism, PPARγ signaling, and triacylglyceride biosynthesis processes (Figure S2). The only process enhanced solely on day 1 is transmembrane receptor protein tyrosine kinase adaptor protein activity. Many pathways are upregulated on day 2 and remain enhanced during the remainder of differentiation. These pathways include cellular alcohol metabolism and cholesterol metabolism. By day three vitamin A metabolism, glucose metabolism, cholesterol biosynthesis, and miR 516a-5p are all upregulated. Interestingly the nuclear envelope-endoplasmic reticulum (ER) network is also enriched in day 2 and day 3 differentiating cells. It is reported that inducing ER stress can suppress adipogenesis [29], and our results confirm this link between the ER and adipogenesis. Overall, these data suggest that OP9 cells undergo a differentiation process similar to other adipocyte models.

### Knock-down of target genes in adipogenesis

After characterizing the transcriptome of OP9 clone K cells, we conclude OP9 adipogenesis is similar to other cell models. The main difference between 3T3-LI cells and OP9 cells is the differentiation period. OP9 cells differentiate in 72 hours enabling screens of transient RNAi and drugs with a short half-life. To test if assessment of gene function on adipogenesis through transient knock-down of target genes is possible in OP9 cells, we evaluated OP9 clone K transfectability, the efficiency of gene knock-down, and the effect of knock-down of genes essential for adipogenic differentiation.

**Figure 3:**
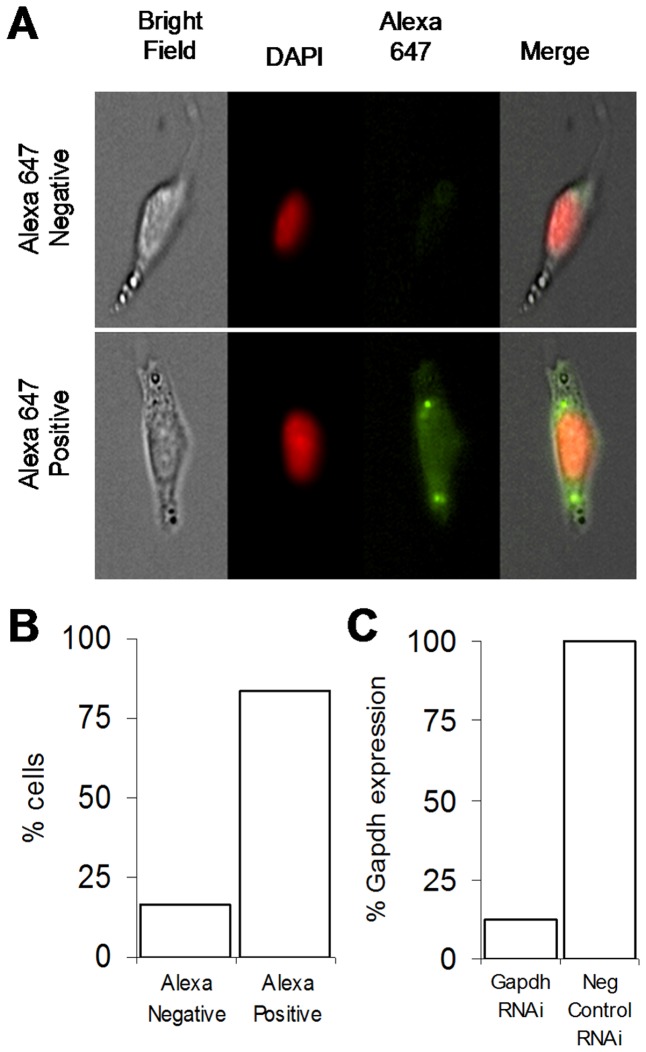
OP9 cells are efficiently and effectively transfected. (A) OP9 cells uptake Alexa (green) tagged RNAi during transfection. Amanis imagestream images of OP9 clone K cells transfected with Alexa 647 tagged RNAi and sorted based on Alexa 647 fluorescence. (B) Quantification of cell populations as classified by Amanis imagestream. (C) Relative expression of GAPDH demonstrates gene knockdown in OP9 cells. qPCR analysis of GAPDH expression level in two GAPDH RNAi samples and two negative control RNAi samples.

To assess if OP9 cells are readily transfectable an Alexa 647 negative control RNAi was transfected into OP9 clone K cells. Cells imaged 48 hours later have green foci, indicating successful transfection (Figure 3a). To quantify the percent of transfected cells we used the Amnis ImageStream, multispectral imaging flow cytometer. Roughly 83.5% of the cells contain green foci (Figure 3b). To study the ability of RNAi to knock-down transcripts in OP9 cells we transfected RNAi directed against GAPDH. We observed an 8-fold reduction in GAPDH expression level in cells transfected with GAPDH RNAi (Fig 3c). These experiments demonstrate OP9-K cells are readily transfectable.

**Figure 4:**
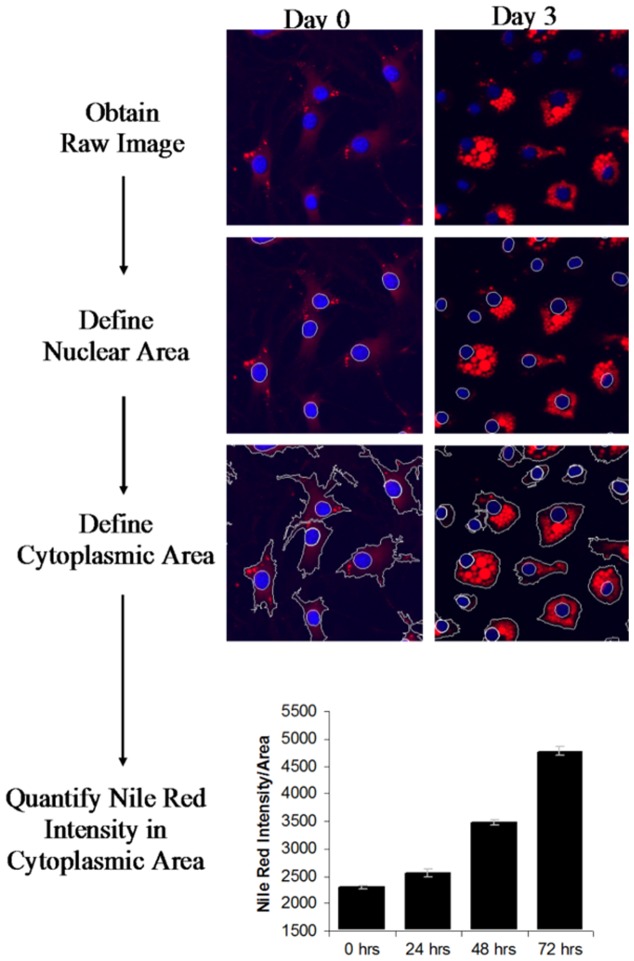
Automated lipid accumulation detection enables high throughput adipogenesis assay. Analysis starts with the raw image as captured by microscopy system. The program draws a boundary around the DAPI foci, identifying the nuclei (shown in blue). A cell outline is drawn around each nucleus, identifying the cell boundary. Lastly, the intensity of Nile Red staining within the cell boundary is quantified (Nile Red shown in red). The analysis steps are shown on representative images of differentiating OP9 clone K cells over the course of 72 hours.

To quantify differentiation in a rapid and reproducible manner, images of differentiating cells were obtained on the ImageExpress system and a lipid analysis scheme was created using MetaXpress. Images of DAPI and Nile Red stained cells were captured. Using MetaXpress software, we created a program to identify the DAPI stained nuclei, expand the region around the nuclei to identify the cell boundaries, and quantify the intensity of Nile Red staining in each cell (Figure 4)(Materials and Methods)(Appendix S1). The system allows us to compare Nile Red staining intensity (threshold NR intensity) of cells with different treatments. Preliminary analysis of differentiating OP9 clone K cells using the Metaxpress software shows a correlation between the integrated intensity measurements and lipid accumulation (Figure 4). This demonstrates that cellular triglyceride accumulation can be measured in a rapid and reproducible manner using automated microscopy analysis.

**Figure 5:**
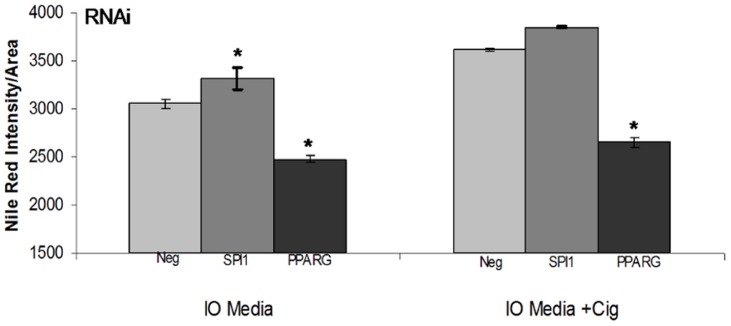
Adipogenesis is increased by RNAi against Spi1 and inhibited by RNAi against Pparg. OP9 clone K cells were transfected with RNAi against the known adipogenic master regulator Pparγ, the transcription factor Spi1, or negative control. Cells were treated with insulin oleate differentiation media with and without the Pparγ agonist, ciglizitone, for 72 hours, imaged using the high-throughput image express system, and Nile Red content quantified using a custom analysis scheme. Intensity values are shown are the average of 72 images. Error bars represent the standard error of the mean. * indicated significantly different from negative control at P<0.05.

To validate our automated analysis scheme, we knocked-down the master adipogenic regulator Pparγ in our OP9 clone K cells (Figure S3). It has previously been demonstrated that Pparγ is necessary for adipogenesis [30]. Our results confirm this finding. In OP9 clone K cells, treatment with RNAi directed against Pparγ prevents the accumulation of lipid in cells grown in IO media for 72 hours (Figure 5, p<0.0001). Therefore we show the power of our gene knockdown analysis assay to detect alterations in lipid accumulation during adipogenesis.

After validating our clonal cell line and automated analysis scheme, we tested the effect of a reduction in Spi1 mRNA, a transcription factor with a potential role in obesity. In OP9 clone K cells, RNAi directed against Spi1 (Figure S4) leads to increased accumulation of lipid in cells grown in IO media for 72 hours (Figure 5). Additional treatment with the Pparγ agonist ciglizitone further increases lipid levels. Reduction of Spi1 levels leads to adipocyte hypertrophy, which is further increased by induction of the Pparg cascade. This indicates a role for Spi1 in the modulation of fat deposition.

## Discussion

We set out to establish OP9 clonal cells as a model of adipogenesis suited for gene-depletion studies of mid and late stage adipogenic genes. The gene expression profiles of differentiating OP9 clone K cells were studied to determine if classical adipogenic marker genes and processes identified in other cell lines are important for OP9 cell differentiation as well. After confirming OP9 clone K cells as a model of adipogenesis, we establish a method for evaluating gene-depletion effects in OP9 cells in mid to late adipogenesis.

The process of lipogenesis and adipogenesis are intimately linked. The gene expression patterns and pathways activated and repressed in OP-K cells upon challenge with insulin and lipids indicate that, at a bare minimum, adipocyte maturation is being triggered [31], [32]. Adipogenic marker gene expression data has led us to conclude that OP9 clone K cells are more committed than the widely used cell model of adipogenesis, 3T3-L1 cells, displaying patterns of mid to late adipogenesis more akin to Ob17 preadipocytes [33]. 3T3, Ob17, and mesenchymal stem cells upregulate C/Ebpβ expression during the early stage differentiation [34]–[36]. C/Ebpβ is highly expressed in OP9 clone K cells as preadipocytes and throughout the differentiation time course. We also show upregulation of the mid and late stage adipogenesis genes C/Ebpα and Plin1 during differentiation. In 3T3 cells C/Ebpa and Plin1 are upregulated during the terminal growth arrest stage, roughly 4 days into the induction phase of 3T3 cell differentiation [37]. C/Ebpα and Plin1 are also upregulated during adipogenesis in human mesenchymal stem cells [38]. Expression of Gata2 inhibits adipogenesis, and is downregulated when 3T3 cells reach confluency after the exponential growth phase [39]. We see this expression pattern mirrored in OP9 cells. As opposed to 3T3-L1, 3T3-F442A and Ob17 cellular models of adigenesis, OP9-K cells treated with insulin oleate media differentiate without reaching confluency or undergoing clonal expansion, and gene expression patterns mirror this difference. Moving forward, it would be interesting to compare the transcriptome of differentiating OP9 preadipocytes with visceral and subcutaneous fat depots to determine which subtype of adipose is represented by OP9 cells.

Adipogenesis involves canonical pathways such as triacylglyceride biosynthesis, PPARγ signaling, and cholesterol metabolism [36], [40]–[43]. Recent literature also suggests that microRNAs play an important role in adipogenesis and obesity [44]–[47]. Several microRNAs have been demonstrated to decrease adipogenesis, including miR-155 which targets C/EBPβ and miR-27/miR-130 which both target PPARγ. Han et al. recently validated miR-340 as a potential negative regulator of the obesity-related gene 11β-Hydroxysteroid dehydrogenase type 1 [48]. Our study shows a decrease in expression of genes targeted by miR-340, miR-590, miR-352, miR-568, miR374, and miR-516 during adipogenesis. This demonstrates that beyond validating canonical adipogenic pathways, analysis of the OP9-K transcriptome points toward novel microRNA targets of adipogenesis.

The shorter duration of differentiation in OP9 cells opens up countless possibilities for new methods of rapid discovery in adipose biology and therapeutics. To enable potential high-throughput studies using OP9-K, a scalable lipid accumulation read-out is required. In our study we utilize high content microscopy as a read-out for cellular lipid content. Several studies have previously reported the use of image-based methods to measure fat accumulation [49]–[54]. Dragunow et al. [49] utilizes various methods of image based analysis and concludes that thresholding is the quickest and most accurate. Therefore we have implemented a thresholding based methodology. Prior studies correlate high content microscopy methods with more conventional measures of adipogenesis, such as triglyceride assays and quantitative PCR, demonstrating the validity of high content microscopy as a read-out for lipid accumulation. Coupled with this, Gasparri et al. [55] emphasizes the critical importance of cell type selection in high content screening. Therefore, our approach which combines a novel clonal cell line together with high content microscopy is a step forward in the field of rapid screening of adipocytes for fat accumulation.

Additionally we used the OP9 model of adipogenesis to demonstrate that reduction in levels of SPI1 stimulates adipose generation. This is consistent with previous data demonstrating inhibition of adipogenesis following SPI1 overexpression [56]. Regulation of SPI1 involves transcript inhibition by a lncRNA, leading to an increase in adipogenesis [56]. SPI1 is expressed in visceral white adipose tissue [57]. Data also demonstrates a link between SPI1 and insulin resistance via inflammatory and antioxidant pathways [58]. Linking SPI1 back to inflammation, in macrophages, adipose differentiation related protein (ADRP) is regulated by SPI1 [59]. Our data also indicates that SPI1 operates in a pathway parallel to PPARγ, as addition of the PPARγ agonist ciglizitone has an additive effect on adipogenesis. Taken together these data indicate that SPI1 could play a role in adipogenesis, and subsequently in the development of obesity.

In conclusion, we have established OP9-K cells as an adipose cell model which can enable rapid discoveries in adipose biology and may be suitable for high-throughput RNAi screens of adipogenesis. We validated our model for RNAi screens of adipogenesis by depleting OP9 cells of Pparγ and demonstrating a resultant inhibition of adipogenesis using high content microscopy. A screen for essential adipogenic genes is important to discover novel therapeutic targets for obesity. Hyperplasia and hypertrophy of adipose cells greatly increases the risk of developing cardiovascular disease [9], [60], [61]. Due to the central role of adipocyte hyperplasia in the pathogenesis of obesity, discovering genes which inhibit or induce adipogenesis provides important drug and therapeutic targets and the OP9-K line will expedite these discoveries.

## Materials and Methods

### RNA interference

Cells were plated in 6-well plates at a density of 3,000 cells/cm2 24 hours prior to transfection. OP9 cells were transfected with RNAi (Silencer Select Pre-designed siRNA, Silencer FAM labeled Negative Control #1 siRNA) using Optifect reagent per the product instructions with 30 nM of target RNAi and 10 uL of optifect reagent in 250 uL of Opti-MEM I Reduced Serum medium (Invitrogen) for each well. The siRNA:lipid transfection complex was added to the cells and incubated overnight. Knock-down was confirmed using qPCR assays specific for each target. *Propagation of OP9 cells*: Parental OP9 cells ([25] and American Type Culture Collection Manassas, VA; catalog no. CRL-2749) were generously provided by Dr. Coleen McNamara (University of Virginia, Charlottesville) [62]. OP9 cells were grown in propagation media consisting of MEM-alpha (Invitrogen, cat# 12571-063) with 20% premium fetal bovine serum, 0.02 mM L-glutamine, and 500 Units of Penicillin Streptomycin. Cells were passaged at 80% confluency by rinsing once with Hank's Balanced Salt Solution, followed by 3 minute incubation with Trypsin-EDTA at 37 degrees C. Cells were passaged at a ratio or 1∶2–1∶4. Cells were frozen in 20% FBS, 5% DMSO, and 75% Propagation Media.

### Differentiation of OP9 cells

Cells were differentiated using Insulin Oleate media. Preparation of insulin oleate media is described by Wolins et al. in detail (18). Briefly, cells were plated at 12,500 cell/cm2 in 96 well plates, and 24 hours later propagation media was replaced with insulin oleate media (MEM-α, 0.2% FBS, 175 nM insulin, 900 µM oleate:albumin, and 500 U penn/strep).

### Triglyceride Assay

Triglyceride assay was performed according to the manufacturer's protocol. The Biovision TG assay (Cat #K622-100) was used in parallel with the Pierce BCA Protein Assay Kit (Thermo Scientific, cat# 23227) for standardization. Cells were lysed in 100 uL of a 5% Triton-x solution in water. The cell/lysis solution was thoroughly mixed to assure total lysis. The plate was subsequently sealed and incubated at 80 degrees C in a water bath for 5 minutes. Plates were then cooled to room temperature and heated in the water bath again. Following lysis, 10 uL of cell lysate was used in the TG assay. TG and protein were measured in triplicate. Significant differences in TG content were determined by two tailed t-test.

### Microscopy (Imaging)

DAPI (Sigma cat# D8417) was diluted in water to 700 uM, and stored at -20 degrees C. Cells were fixed cells for 20 minutes at room temperature in 4% Paraformaldehyde (PFA). Pipetting steps were performed gently, as differentiated adipocytes are easily dislodged. PFA was removed and replaced with 100 uL of 2 uL/mL of stock DAPI in 1∶1 glycerol:PBS and incubated for 5 minutes at RT. DAPI stain was removed and 100 uL of a 1∶10000 Nile Red (Sigma) solution in PBS added (prepared from 1 mg/ml stock) and incubated for a minimum of 25 minutes at RT. Cells were imaged in the nile red solution. Conventional microscopy was performed for initial low throughput studies of clonal differentiation. To determine the percent of cells containing lipid droplets, using 40x magnification, 8 fields of cells were visually counted for the presence of Nile-red stained lipid droplets. For high content microscopy, plates were imaged in the ImageExpress robotic microscope in the core facility. Images were acquired at 40x magnification across 16 sites per well with 12 wells per condition. The image analysis is performed with a customized journal with MetaXpress, chaining together image analysis steps. The first steps involve cell segmentation using MetaXpress built-in "Cell Scoring" functionality with DAPI and NileRed. We determined the total number of cells per image, and the cell segmentation is also achieved in this step. Using the cell segmentation as a mask, the area of each cell was determined. The integrated NileRed staining intensity (including oil droplet and other lipid component, such as membrane lipid) for each cell was determined. A threshold was then applied to the NileRed image to separate the dimmer cellular membrane or lipid NileRed stain from the bright oil droplets. The area and integrative NileRed intensity of oil droplets with each cell was then measured. The resulting measurement was then summarized and analyzed in AcuityXpress. Specific details about threshold and other journal steps can be seen in Appendix S1.

### Quantitative PCR

cDNA was generated using the Applied Biosystems TaqMan Reverse Transcription Reagents (P/N N808-0234) according to the manufacturer's protocol. Quantitative PCR reactions were performed in 10 uL in 384 well plates using 2x sybr green, 50 nM forward primer, 50 nM reverse primer, 5 ng cDNA, and 3 uL of water. Reactions were cycled at 95 degrees for 10 min, followed by 40 cycles of 95 degrees for 15 seconds and 60 degrees for 1 min. Data were analyzed using the relative quantification delta delta Ct method. For each sample, six biological and three technical replicates were used. Significant differences in data were calculated using a two-tailed t-test.

### RNA extraction

RNA was extracted using the Ambion Ribopure kit (Ambion, cat# AM1924). The manufacturer's protocol was followed with the following exception: the phenol extraction step was performed on 15 mL heavy phase lock gel according to the manufacturer's protocol (5Prime, cat# 2302850). The organic and inorganic phases were separated on phase lock gel, and the organic phase was collected, and the Ribopure protocol subsequently followed.

### Generation of clonal cell lines

The OP9 parental cell line was trypsinized and resuspended at a density of.5 cells/100 uL of propagation media. 100 uL of cell suspension was plated in each well of a 96 well plate. Plates were checked every 48 hours for the appearance of visible colonies. Colonies were subsequently tested using the differentiation assay described above.

### Microarray analysis

Total RNA was extracted from three biological replicates for each differentiation timepoint as described above and hybridized to Affymetrix Mouse Gene 1.0 ST Arrays. Data were analyzed using Expression Console from Affymetrix and the R Limma package. Microarray data was normalized using the Robust Multichip Average (RMA). Data was filtered at a cutoff of 3.5, the background threshold for the mouse ST 1.0 array. Fold change data, p-values, and FDR adjusted p-values were generated by the R limma function “TopTable”. Gene ontology overrepresentation was calculated using g:Profiler (http://biit.cs.ut.ee/gprofiler/). Microarray probes were sorted by fold change for each time point and the 250 genes with the largest positive fold change, and the 250 genes with the largest negative fold change were selected for analysis. G:Profiler was run using default parameters, organism (*mus musculus*), and probe IDs as input.

## Supporting Information

Table S1 **Top 25 overexpressed genes at each time point relative to 0 hrs.**
(XLS)

Table S2 **Top 25 down regulated genes at each time point relative to 0 hrs.**
(XLS)

Figure S1 **OP9-K adipogenesis involves down-regulation of biological processes common to adipogenesis.** Functional profile of the 250 genes with the greatest fold change decrease during OP9 adipogenesis as identified using GProfiler. As demonstrated in previous models, osteogenesis and cell cycle processes are down-regulated. The transcriptome of OP9 adipogenesis is similar to previously characterized adipocyte models.
(PPT)

Figure S2 **OP9-K adipogenesis involves up-regulation of biological processes common to adipogenesis.** Functional profile of the 250 genes with the greatest fold increase genes during OP9 adipogenesis as identified using GProfiler. As demonstrated in previous models, PPARγ signaling is induced and triacylglyceride is synthesized. The transcriptome of OP9 adipogenesis is similar to previously characterized adipocyte models.
(PPT)

Figure S3 ***Pparγ*** **levels are significantly reduced in OP9-K cells after treatment with RNAi against Pparγ.** Expression levels were measuring using relative quantification RT-PCR with biological and technical replicates. Expression levels are shown relative to OP9-K cells treated with negative control RNAi. p-value represents the significance level of knock down as calculated with a t-test. Error bars represent the 95% confidence interval.
(PPT)

Figure S4 ***Spi1*** **levels are significantly reduced in OP9-K cells after treatment with RNAi against Spi1.** Expression levels were measuring using relative quantification RT-PCR with biological and technical replicates. Expression levels are shown relative to OP9-K cells treated with negative control RNAi. p-value represents the significance level of knock down as calculated with a t-test. Error bars represent the 95% confidence interval.
(PPT)

Appendix S1 **Journal Code for MetaXpress.**
(DOCX)

File S1 **Microarray Cel files for OP9-K day 0 and day 1 for biological replicates 0-3.**
(ZIP)

File S2 **Microarray Cel files for OP9-K day 2 and day 3 for biological replicates 0-3.**
(ZIP)
