# Supplementary material for: Development of an OP9 Derived Cell Line as a Robust Model to Rapidly Study Adipocyte Differentiation
Source: PLoS One. 2014 Nov 19;9(11):e112123. doi: 10.1371/journal.pone.0112123 (PMC4237323; doi:10.1371/journal.pone.0112123)
Supplement: Figure S1 — OP9-K adipogenesis involves down-regulation of biological processes common to adipogenesis. Functional profile of the 250 genes with the greatest fold change decrease during OP9 adipogenesis as identified using GProfiler. As demonstrated in previous models, osteogenesis and cell cycle processes are down-regulated. The transcriptome of OP9 adipogenesis is similar to previously characterized adipocyte models. (PPT) [file pone.0112123.s003.ppt]

## Slide 1
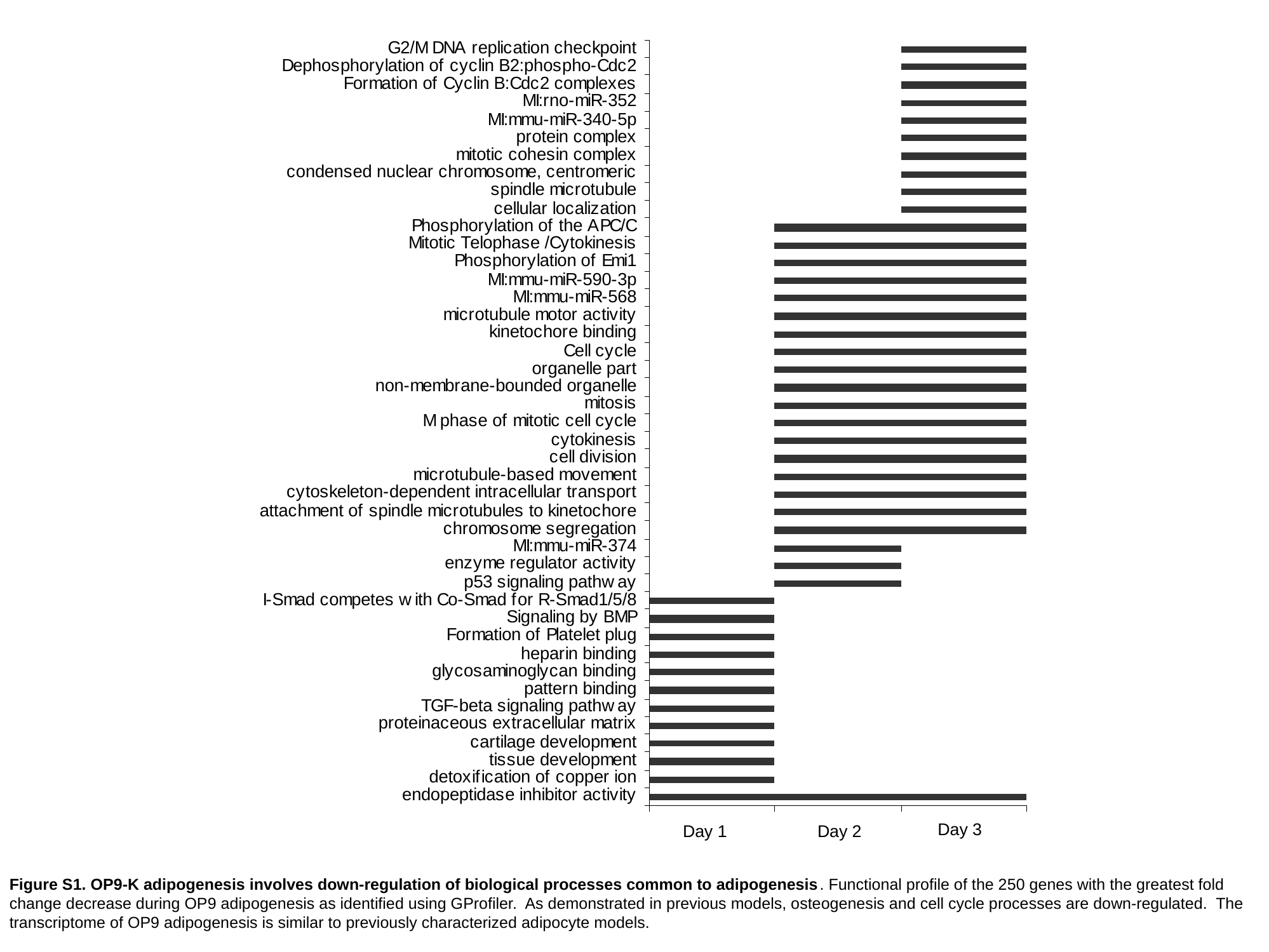

Figure S1. OP9-K adipogenesis involves down-regulation of biological processes common to adipogenesis. Functional profile of the 250 genes with the greatest fold change decrease during OP9 adipogenesis as identified using GProfiler. As demonstrated in previous models, osteogenesis and cell cycle processes are down-regulated. The transcriptome of OP9 adipogenesis is similar to previously characterized adipocyte models.
