# Supplementary material for: Development of an OP9 Derived Cell Line as a Robust Model to Rapidly Study Adipocyte Differentiation
Source: PLoS One. 2014 Nov 19;9(11):e112123. doi: 10.1371/journal.pone.0112123 (PMC4237323; doi:10.1371/journal.pone.0112123)
Supplement: Figure S2 — OP9-K adipogenesis involves up-regulation of biological processes common to adipogenesis. Functional profile of the 250 genes with the greatest fold increase genes during OP9 adipogenesis as identified using GProfiler. As demonstrated in previous models, PPARγ signaling is induced and triacylglyceride is synthesized. The transcriptome of OP9 adipogenesis is similar to previously characterized adipocyte models. (PPT) [file pone.0112123.s004.ppt]

## Slide 1
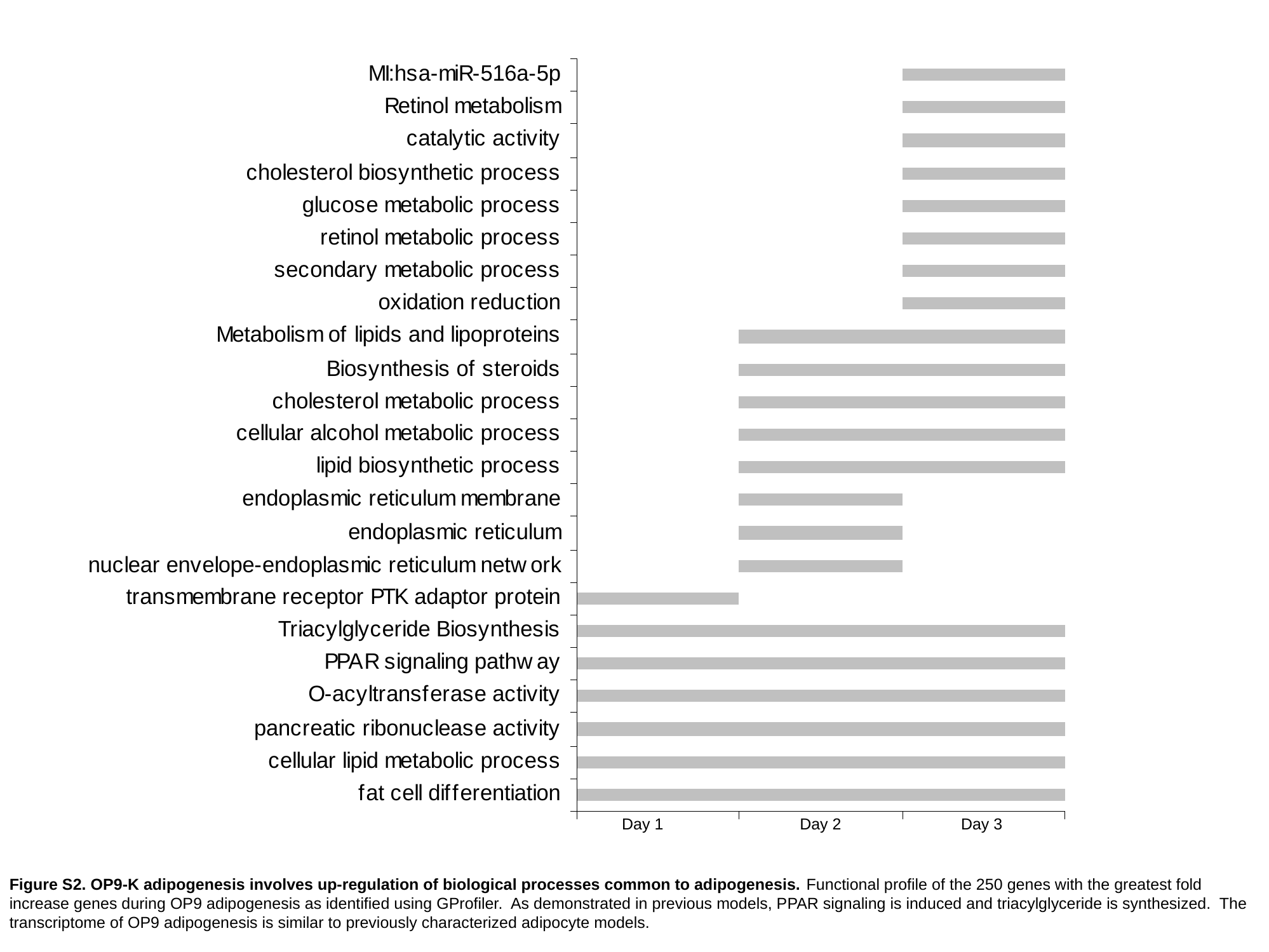

Day 1
Day 2
Day 3
Figure S2. OP9-K adipogenesis involves up-regulation of biological processes common to adipogenesis. Functional profile of the 250 genes with the greatest fold increase genes during OP9 adipogenesis as identified using GProfiler. As demonstrated in previous models, PPAR signaling is induced and triacylglyceride is synthesized. The transcriptome of OP9 adipogenesis is similar to previously characterized adipocyte models.
