# Supplementary material for: Development of an OP9 Derived Cell Line as a Robust Model to Rapidly Study Adipocyte Differentiation
Source: PLoS One. 2014 Nov 19;9(11):e112123. doi: 10.1371/journal.pone.0112123 (PMC4237323; doi:10.1371/journal.pone.0112123)
Supplement: Figure S3 — Pparγ levels are significantly reduced in OP9-K cells after treatment with RNAi against Pparγ. Expression levels were measuring using relative quantification RT-PCR with biological and technical replicates. Expression levels are shown relative to OP9-K cells treated with negative control RNAi. p-value represents the significance level of knock down as calculated with a t-test. Error bars represent the 95% confidence interval. (PPT) [file pone.0112123.s005.ppt]

## Slide 1
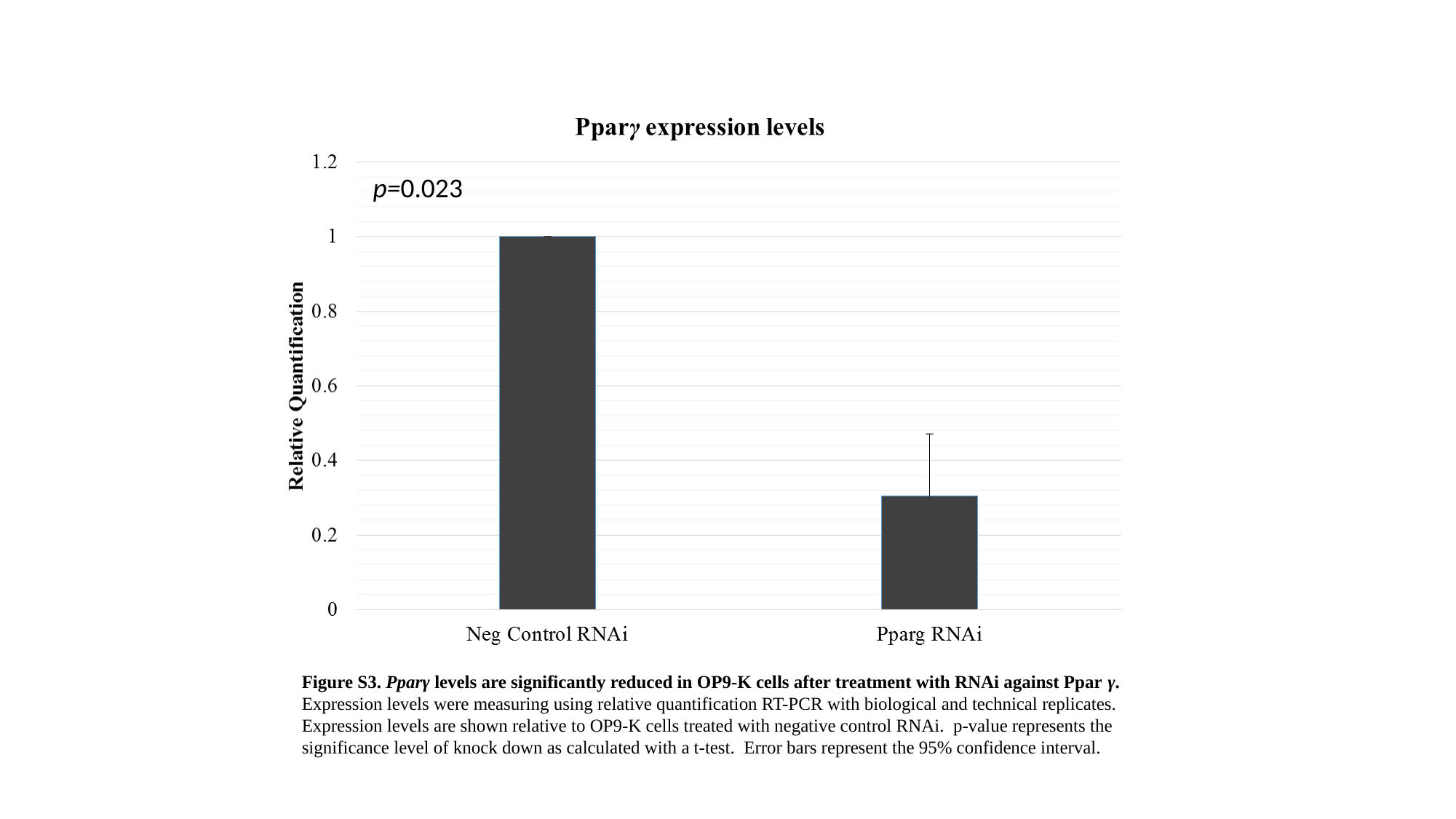

p=0.023
Figure S3. Pparγ levels are significantly reduced in OP9-K cells after treatment with RNAi against Ppar γ. Expression levels were measuring using relative quantification RT-PCR with biological and technical replicates. Expression levels are shown relative to OP9-K cells treated with negative control RNAi. p-value represents the significance level of knock down as calculated with a t-test. Error bars represent the 95% confidence interval.
