# Supplementary material for: Development of an OP9 Derived Cell Line as a Robust Model to Rapidly Study Adipocyte Differentiation
Source: PLoS One. 2014 Nov 19;9(11):e112123. doi: 10.1371/journal.pone.0112123 (PMC4237323; doi:10.1371/journal.pone.0112123)
Supplement: Appendix S1 — Journal Code for MetaXpress. (DOCX) [file pone.0112123.s007.docx]

Journal for DAPI/Nile Red Analysis

1: Open Summary Log(OPENDDE and OVERWRITEMODE, "")

Using Cell Scoring App to segment cells and records total cell number using nuclear stain

2: Overwrite "Segmentation" = Cell Scoring(All nuclei = "DAPI - Pos 1", Positive marker = "TRITC - Pos 4") TotalCellNumber = CellScoring.TotalCells Threshold bright Nile Red staining/droplets and measured their area and integrated intensity of the dye

3: Overwrite "Multiply" = ("TRITC - Pos 4" * "Segmentation") / 1

4: Threshold Image("Multiply", 1, 65535)

5: Show Region Statistics("Multiply", ENTIREIMAGE)

TotalCellArea = ShowRegionStatistics.ThresholdedArea

WholeCellInetgratedIntensity = ShowRegionStatistics.Integrated

6: Threshold Image("Multiply", 750, 65535)

7: Show Region Statistics("Multiply", ENTIREIMAGE)

ThresholdedNileRedArea = ShowRegionStatistics.ThresholdedArea

ThresholdedNileRedIntegratedIntensity = ShowRegionStatistics.Integrated

Reporting measurement to Excel spreadsheet

8: Log Variable(TotalCellArea, NONEWLINE, NO HEADER)

9: Log Variable(WholeCellInetgratedIntensity, NONEW LINE, NO HEADER)

10: Log Variable(ThresholdedNileRedArea, NONEWLINE, NO HEADER)

11: Log Variable(ThresholdedNileRedIntegratedIntensity, NONEWLINE, NO HEADER)
